# Ceruloplasmin and Hypoferremia: Studies in Burn and Non-Burn Trauma Patients

**DOI:** 10.3390/antiox4010153

**Published:** 2015-03-06

**Authors:** Michael A. Dubick, Johnny L. Barr, Carl L. Keen, James L. Atkins

**Affiliations:** 1 U.S. Army Institute of Surgical Research, 3698 Chambers Pass, JBSA Fort Sam Houston, TX 78234, USA; E-Mail: Johnny.l.barr.civ@mail.mil; 2 Departments of Nutrition and Internal Medicine, University of California, Davis, CA 95616, USA; E-Mail: clkeen@ucdavis.edu; 3 Walter Reed Army Institute of Research, Silver Spring, MD 20910, USA; E-Mail: jim.atkins.w@gmail.com

**Keywords:** ceruloplasmin, ferroxidase, iron status, oxidant stress, burn, trauma

## Abstract

**Objective**: Normal iron handling appears to be disrupted in critically ill patients leading to hypoferremia that may contribute to systemic inflammation. Ceruloplasmin (Cp), an acute phase reactant protein that can convert ferrous iron to its less reactive ferric form facilitating binding to ferritin, has ferroxidase activity that is important to iron handling. Genetic absence of Cp decreases iron export resulting in iron accumulation in many organs. The objective of this study was to characterize iron metabolism and Cp activity in burn and non-burn trauma patients to determine if changes in Cp activity are a potential contributor to the observed hypoferremia. **Material and Methods**: Under Brooke Army Medical Center Institutional Review Board approved protocols, serum or plasma was collected from burn and non-burn trauma patients on admission to the ICU and at times up to 14 days and measured for indices of iron status, Cp protein and oxidase activity and cytokines. **Results**: Burn patients showed evidence of anemia and normal or elevated ferritin levels. Plasma Cp oxidase activity in burn and trauma patients were markedly lower than controls on admission and increased to control levels by day 3, particularly in burn patients. Plasma cytokines were elevated throughout the 14 days study along with evidence of an oxidative stress. No significant differences in soluble transferrin receptor were noted among groups on admission, but levels in burn patients were lower than controls for the first 5 days after injury. **Conclusion**: This study further established the hypoferremia and inflammation associated with burns and trauma. To our knowledge, this is the first study to show an early decrease in Cp oxidase activity in burn and non-burn trauma patients. The results support the hypothesis that transient loss of Cp activity contributes to hypoferremia and inflammation. Further studies are warranted to determine if decreased Cp activity increases the risk of iron-induced injury following therapeutic interventions such as transfusions with blood that has undergone prolonged storage in trauma resuscitation.

## 1. Introduction

Iron is an essential element for life that facilitates important biologic redox reactions critical for survival such as those involved in oxygen transport and cellular respiration. However, such properties can also lead to untoward effects as iron can catalyze reactions with oxidants through the Fenton Reaction, for example, to produce highly reactive and damaging radicals such as the hydroxyl radical. To minimize deleterious effects of iron while providing it to necessary sites within the body, the systemic transport, handling and distribution of iron is normally highly regulated [1,2,3]. Although research in this area has provided mechanisms related to most common iron disorders [4,5], much remains to be elucidated about the effects of transient alterations in iron homeostasis that can occur in critically-ill patients [6,7].

Previous studies have reported a disruption of normal iron handling in critically ill patients that may contribute to systemic inflammation and distant organ injury [8,9]. Hypoferremia is a common finding in such patients [10,11,12], but mechanism(s) responsible have not been fully identified. Similar hypoferremia is seen after the infusion of endotoxin into healthy volunteers [13] that may relate to secretion of hepcidin, a peptide that induces the degradation of ferroportin, the only known exporter of cellular iron [14], although other mechanisms may be involved [15]. Recently, elevated levels of hepcidin were observed in trauma patients [16]. A possible mechanism for this hypoferremia is a decrease in the activity of ceruloplasmin (Cp), a multi-copper oxidase that possesses ferroxidase and antioxidant activity. It is important in the conversion of ferrous iron secreted by ferroportin, into ferric iron that can bind transferrin. Lack of Cp results in the degradation of ferroportin. As an acute phase protein, plasma Cp levels typically rise in inflammatory states [17,18,19], but early time points of Cp activity have not been carefully examined.

The first evidence of changes in Cp in trauma came in an animal study of hemorrhagic shock in which the electron spin resonance signature of Cp was significantly reduced [8]. In order to evaluate the possibility that changes in Cp could potentially contribute to the early hypoferremia in burn and trauma patients the current study characterized Cp protein levels and oxidase activity in burn and non-burn trauma patients during their hospital course in the USAISR Burn Unit or Brooke Army Medical Center Intensive Care Unit (BAMC ICU), as well as changes in the iron status of these patients.

## 2. Materials and Methods

This paper is based on observations in two separate studies, both approved by the Brooke Army Medical Center Institutional Review Board and in accordance with the approved protocols. The first study was a preliminary investigation of iron and other trace element levels, and select indices of oxidative stress, in serum from 10 burn patients on admission to the US Army Burn Unit (day 1) and on days 3, 7 and 14. The second study was approved as an addendum under a larger protocol examining coagulation and immunological status in burn and non-burn trauma patients during their hospital course [20]. For both studies, patients or their families were given an information sheet describing this less than minimal risk study before being consented and enrolled. Patients were admitted to the US ArmyBurn Unit or BAMC ICU within 24 h of injury. Blood was collected at the time of phlebotomy on 24 burn and 35 non-burn trauma patients on admission to the ICU (within 24 h of injury; baseline) and at days 1, 3, 5, 7 and 14 as available. Blood was also collected under informed consent from 20 healthy subjects and their laboratory data used as reference controls. Patients were assessed for Injury Severity Score (ISS) and Percent Total Body Surface Area burn, where appropriate. Other details of the protocol have been described [20].

In the first study, serum was obtained at the time of admission (day 0) and assayed for iron levels, unsaturated iron binding capacity and total iron binding capacity using commercial kits. Serum transferrin, total soluble transferrin receptor and ferritin concentrations were also determined by commercial kits. Serum IL-1β levels were determined using the R & D Systems kit (R & D, Minneapolis, MN, USA). Serum uric acid concentrations were determined by standard clinical chemistry assay. Glutathione peroxidase activity was determined as described by Lawrence and Burk [21] and assay of serum total antioxidant potential was as described by Benzie and Strain [22]. In addition, serum total iron, copper and zinc were determined by inductively coupled axial plasma spectroscopy [23].

In the second study, Cp protein levels were determined by a commercial ELISA kit (Assaypro, St Charles, MO, USA). Cp oxidase activity was determined spectrophotometrically at 550 nm by a modification of the assay described by Macintyre *et al.* [24] using *N*,*N*-dimethyl-*p*-phenylenediamine as substrate. Soluble transferrin receptor in plasma, as a marker of iron status, was assayed by a commercial kit (Quantikine^®^ IVD^®^ immunoassay (R & D Systems, Minneapolis, MN, USA). Missing data at day 14 or 21 reflect insufficient sample for the assay or patient discharge.

### Statistical Analysis

Data are presented as mean ± SE and were analyzed by repeated measures Analysis of Variance. In study 1, data are presented to denote reference normal ranges when available, since no normal control samples were available for comparison. Where appropriate, results were compared with normal control values by Student’s *t*-test. A *p* < 0.05 was considered significant.

## 3. Results

**Figure 1:**
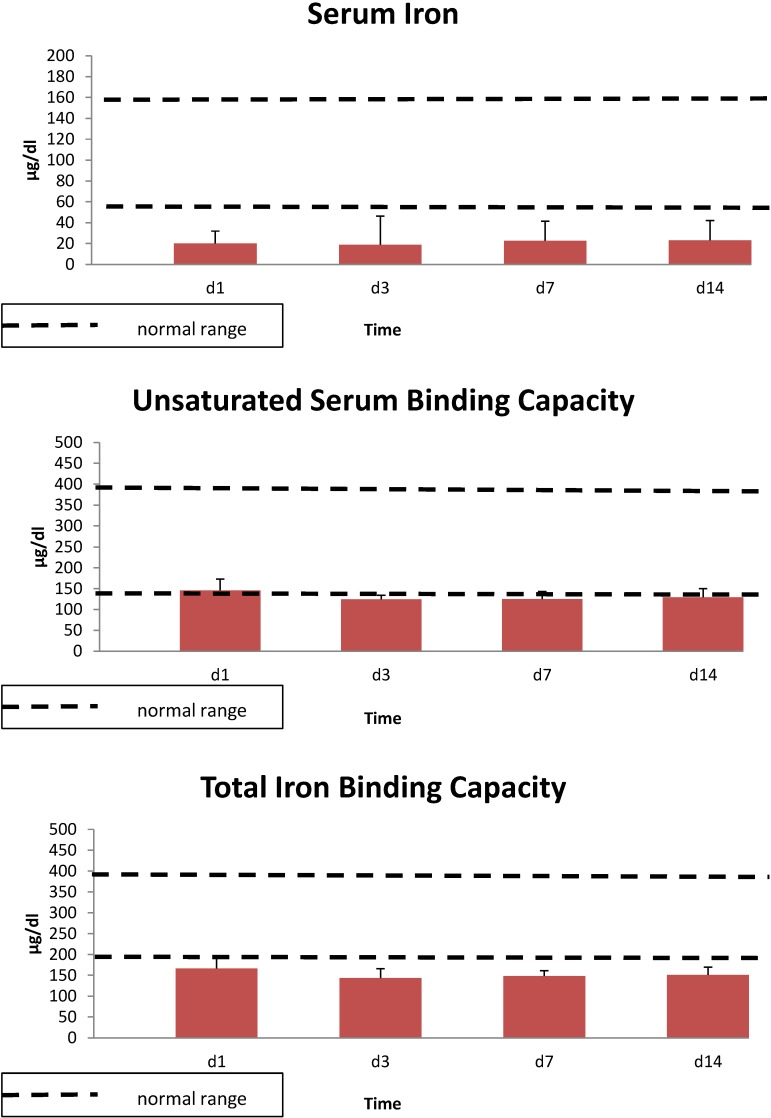
Serum iron (Fe^+2^), unsaturated iron binding capacity and total iron binding capacity over 14 days in 10 thermally injured patients. Data expressed as mean ± SE. Dotted lines denote the upper and lower normal limits.

**Figure 2:**
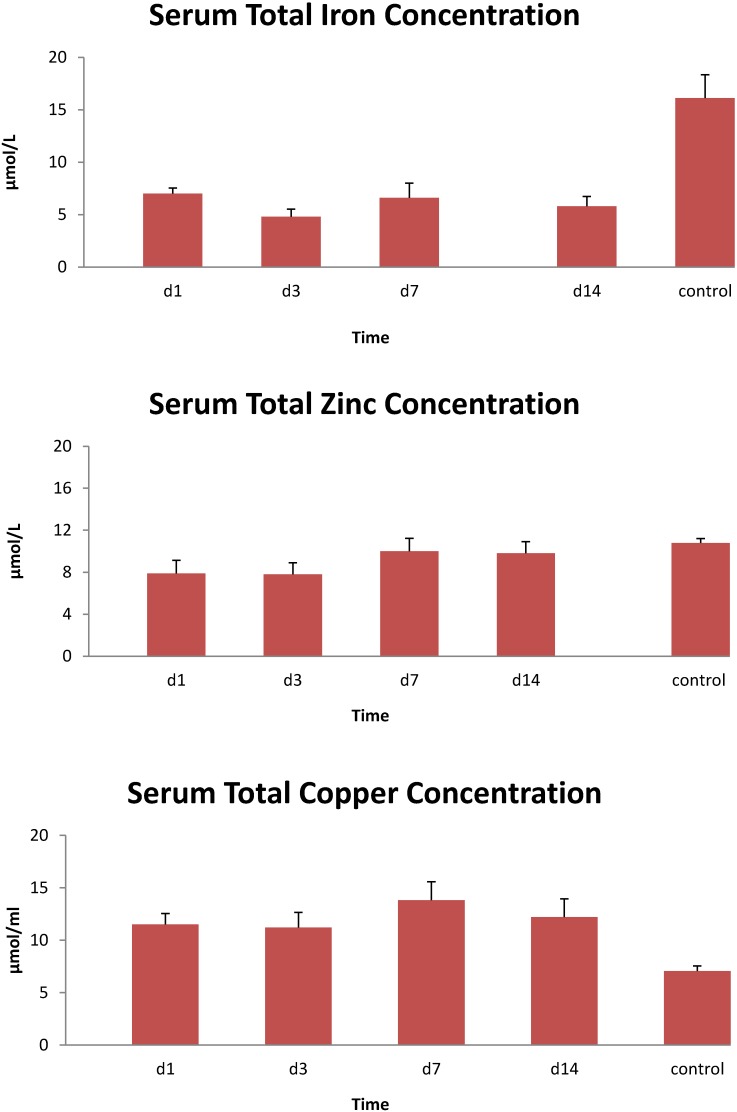
Total serum iron, zinc and copper concentrations in 10 thermally injured patients. Data expressed as mean ± SE. Control data were from historic normal subjects.

**Figure 3:**
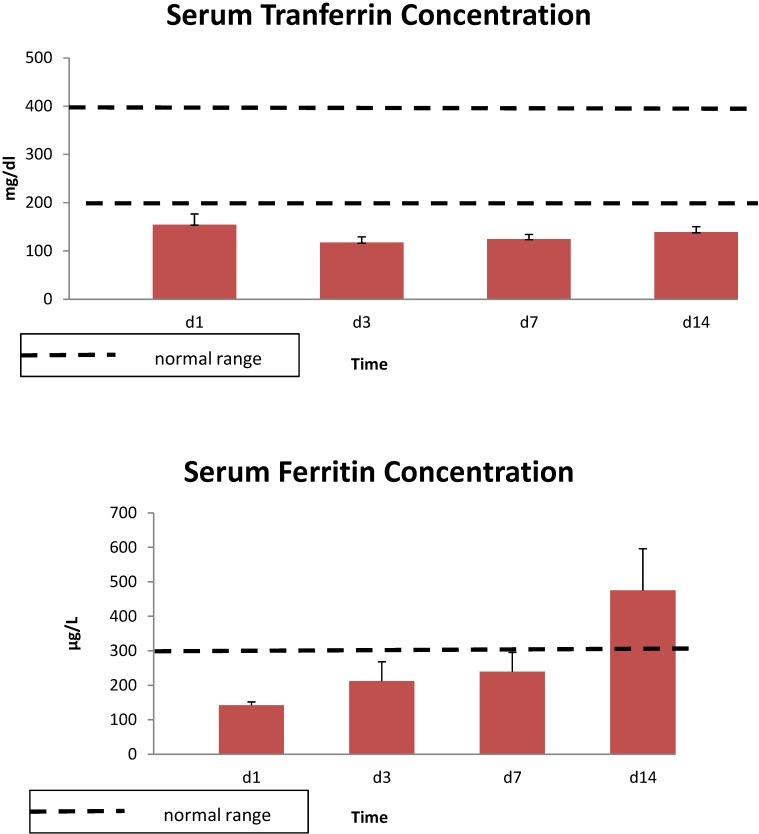
Serum transferrin and ferritin concentrations in 10 thermally injured patients. Data expressed as mean ± SE. Dotted lines denote the upper and lower normal limits.

The demographics of the burn patients in study one are shown in Table 1. All 10 patients were male. Serum iron levels were lower than the normal range on day 1, and values remained low throughout the 14 day study (Figure 1). Similar results were seen with total serum iron levels, while serum zinc concentrations were lower than controls for the first 3 day and copper concentrations were elevated relative to controls throughout the study (Figure 2). Unsaturated iron binding capacity was at the low range of normal throughout the 14 day study, whereas mean total iron binding capacity was 20%–30% below normal levels (data not shown). Average serum transferrin concentrations were below normal levels for the 14 day, whereas ferritin concentrations were largely within normal ranges or exceeded these levels at 14 day (Figure 3). Serum soluble transferrin receptor levels were below the lower limit considered normal at days 1 and 7, but increased to within the normal range by 14 day (data not shown).

**Figure 4:**
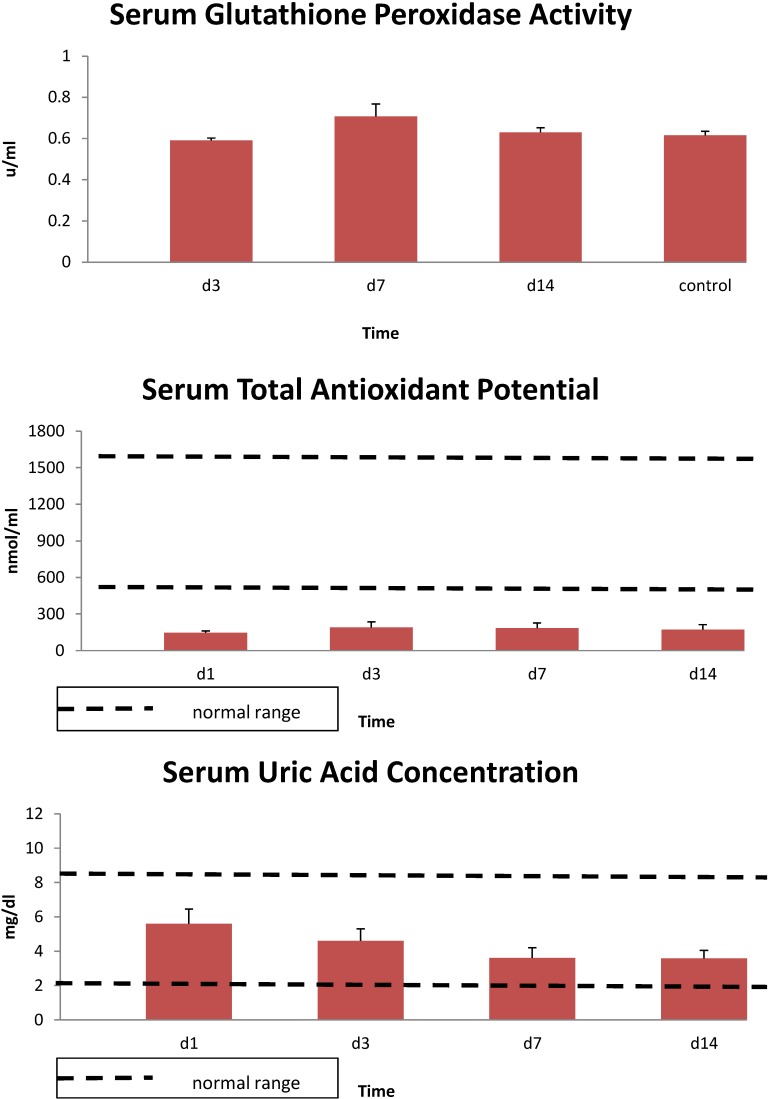
Serum glutathione peroxidase activity, Fe^+2^ reducing potential and uric acid concentrations in 10 thermally injured subjects. Data expressed as mean ± SE. Dotted lines denote the upper and lower normal limits.

In study 1, IL-1β levels were elevated throughout the 14 day study period averaging about 20 ± 5 pg/mL over this period. Although serum total antioxidant potential was lower than control values throughout, glutathione peroxidase activity and uric acid levels were within the control range (Figure 4).

In the second study, the mean age of control subjects was 37 years compared to 56 and 43 years in burn and non-burned trauma patients, respectively. The ISS was 18.1 ± 2.3 and 21.7 ± 1.7 for burn and trauma patients, respectively, and there were no statistical differences between the 2 patient groups. Mean burn size in burn patients was 30% ± 4%, which is similar to that seen in study 1. ICU days were twice as long in the burn than non-burn trauma patients (26.3 ± 6.2 *vs.* 13.0 ± 2.5 days, respectively). ICU days in burn patients were similar in the two studies.

**Figure 5:**
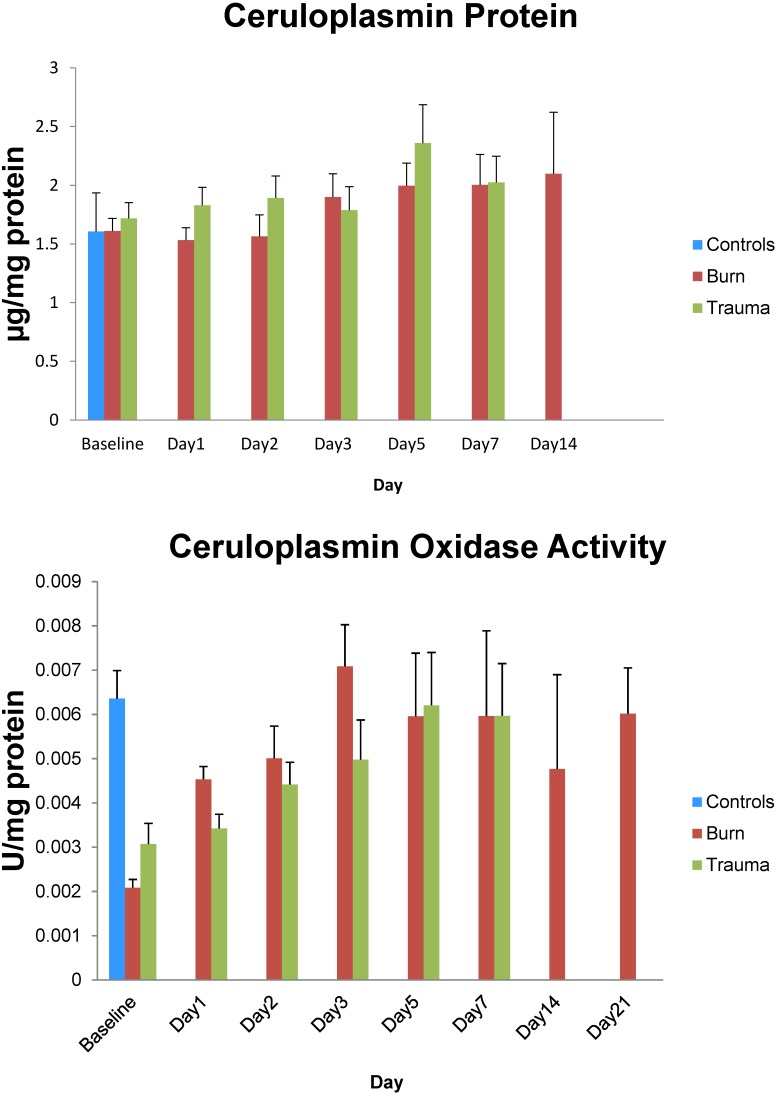
Ceruloplasmin protein concentrations and oxidase activity over 14 days in thermally injured and non-burned trauma patients. Data expressed as mean ± SE from 24 burned and 35 non-burned trauma patients and 10 healthy controls.

In both groups of trauma patients, plasma ceruloplasmin (Cp) protein levels on admission were similar to those in healthy controls (Figure 5). These levels tended to rise over the patients’ courses of hospital stay and were significantly higher than control levels from days 5 to 14 in both groups of patients. In contrast, plasma Cp oxidase activity in burn and non-burn trauma patients was significantly lower than control values on admission (Figure 5). This enzyme activity in both groups of patients recovered to control levels by days 3–5.

**Figure 6:**
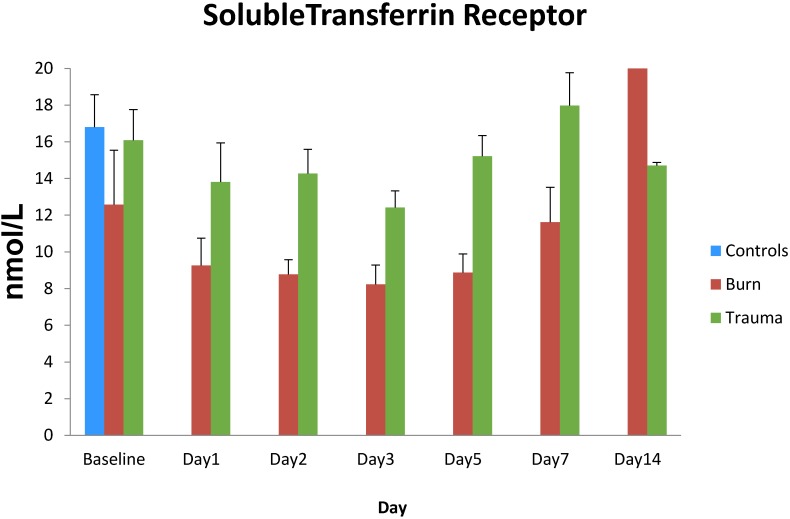
Soluble transferrin receptor levels in plasma from thermally injured and non-burned trauma patients. Data expressed as mean ± SE from 24 burned and 35 non-burned trauma patients and 10 healthy controls.

Soluble transferrin receptor, as an index of iron status, was 35%–50% lower in plasma from the trauma patients than control levels on admission (*p* < 0.05), and was further reduced over the next 5 days in burn patients (Figure 6). Soluble transferrin receptor levels were less affected over time in trauma patients and recovered to admission levels by day 7. Both patient groups returned to near normal control levels at day 14 after admission.

**Table 1:** Demographics of Burn Patients.

Gender: Male *n* = 10, Female *n* = 0
Age: Range 20–50 years; Mean ± SE: 36.8 ± 3.0 years
Total Body Surface Area Burn: Range 19%–76%; Mean ± SE: 36.8% ± 5.3%
Total Full Thickness Burn: Range 0%–56%; Mean ± SE:20% ± 5.7%
Total ICU Time: Range 9–42 days; Mean ± SE: 24.9 ± 4.1 days

## 4. Discussion

The present study provides additional evidence of low serum/plasma iron concentrations in thermally injured patients and the prevalence of anemia in critically injured patients [10,12]. In addition, we observed low iron binding capacity, serum transferrin concentrations and soluble transferrin receptor levels below control levels for at least a week after burn injury, consistent with the report by Berlin *et al.* [11] that soluble transferrin receptor is a good indicator of iron deficiency anemia in hospitalized patients with acute illness. In the current study, soluble transferrin receptor was significantly lower in burn patients than trauma patients until day 14. The low iron status in critically ill or injured patients has been termed “stress hypoferremia” that appears to be independent of injury severity, blood transfusions, need for surgery or development of sepsis [6], although others have reported a link between iron status and injury severity [7,25]. Interestingly, efforts to treat this anemia with intravenous iron supplementation have been unsuccessful to the point that such therapy is not recommended [12], suggesting that factors in addition to simple iron deficiency are involved in the development of this anemia.

These changes are similar to those seen in the hypoferremia of inflammation which has been ascribed to the cytokine-induced release of hepcidin [15,16]. It is postulated that this response evolved from evolutionary pressure to survive blood-borne infections by limiting the availability of iron which is an essential nutrient for most infectious organisms [26]. For example labile iron pool in liver has been shown to increase acutely in hemorrhagic shock [27,28], indicating, moreover, that the liver may be particularly susceptible to iron induced injury in hemorrhagic shock.

The risk of iron-mediated hepatic injury would be expected to be even greater if iron export was inhibited at a time when the blood level of non-transferrin bound iron (NTBI) was increased, as NTBI is avidly taken up by the liver [29,30]. At least one common therapeutic intervention, the transfusion with blood that has undergone prolonged storage, has been shown to lead to increased NTBI [31]. Moreover, it is possible that a decrease in the ferroxidase activity of Cp could directly contribute to the production of NTBI as Cp is normally required to convert ferrous iron to the ferric form that is required for binding to transferrin. If this speculation is correct it would suggest that ideally blood with shorter storage time should preferentially be used when Cp activity is low. Alternatively possibly active Cp could be infused at the time of transfusion. Interestingly, preliminary reports from Russia purport the benefit of Cp infusion in patients who have had severe hemorrhage [32].

Further, the available data suggest that oxidative stress associated with burn and trauma, including inhalation injuries, results in an increase in potentially damaging “labile iron” or NTBI, an observation that is consistent with reports of benefits reported after administration of iron chelators [33,34]. Burn injury often results in hemolysis which releases iron into the extracellular compartment and may contribute to local and distant organ injury, such as to the kidney and lung [35]. The lung has also been cited as being particularly susceptible to iron-mediated injury as many forms of inhalation injury are also improved by iron chelators [33,35]. In addition, a transient increase in Fe^+2^ was observed in mesenteric lymph after hemorrhage, a key observation since both hemorrhage and mesenteric lymph have been linked to development of lung injury [8]. Together with the data from the present studies, it would seem that the ability of Cp to inactivate reactive iron (NTBI) or iron-induced oxidative stress is reduced after trauma, despite normal Cp protein levels. If Cp has a critical role in iron regulation, without other controls, reduced Cp activity would be expected to result in elevated NTBI.

Serum ferritin concentrations were in the normal range for at least the first week after burn injury and increased above normal by day 14, while soluble transferrin receptor was below normal for the first five days after burn and tended to rise toward normal levels at day 14. The observed low transferrin concentrations are in agreement with other studies in trauma or other critically ill patients [6,36,37], whereas reports on ferritin levels are more variable. Although we observed ferritin levels in the burn patients to be in the normal range and then increase above normal by day 14, others have reported elevated ferritin early after acute injury, surgery or illness in both experimental animals and humans [36,37,38,39]. Efforts to understand the ferritin response to injury have focused on development of some form of acute lung injury. In experimental animals, elevated ferritin levels were related to development of lung injury after hemorrhage and this response was not seen in rats fed an iron-deficient diet [39]. In addition, initial ferritin levels in trauma patients were correlated with development and progression of lung injury and injury severity scores, but did not correlate with the number of ventilator days or mortality [9]. Although several stimuli may be responsible for the decrease in soluble transferrin receptor and the increase in ferritin, both are expected responses to an increase in intracellular iron [9]. Increased intracellular ferritin provides additional binding capability for iron and decreased transferrin receptor limits the uptake of transferrin bound iron. These regulated intercellular responses are normally mirrored in the changes in plasma ferritin and plasma soluble transferrin receptor. Thus, the changes in ferritin and soluble transferrin receptor are consistent with an increase in intracellular iron.

The present cytokine data also support an inflammatory response associated with burn and traumatic injuries. We observed significant elevations in IL-1β, IL-6 and IL-8 in both groups of patients [40], which has been commonly reported. The cytokine response may in part be responsible for the increase in hepcidin seen in trauma patients.

In the current study, plasma Cp protein levels in burn and trauma patients were similar to healthy controls on admission to the hospital and levels remained about the same or slightly increased after day 5. This varies somewhat with the observation that total serum copper levels remained relatively constant throughout the 14 day observation period in burn patients, although the levels were significantly higher than normal controls. This suggests that Cp as a marker of copper status may be altered in the critically ill. Nevertheless, Cp oxidase activity was significantly lower than healthy controls in both burn and non-burn trauma patients on admission and activity remained lower than controls for the first 3 days after injury in both groups of patients. To our knowledge this is the first study to measure both Cp enzyme activity and protein levels in the same patients. In addition, to get a more complete picture of the relationship between Cp and iron, serum copper and zinc levels were also measured in the present study. Under homeostatic conditions, iron and copper metabolism are interrelated, as are copper and zinc metabolism [41,42,43]. Serum copper and zinc serum concentrations were not predictive of the present Cp data. It should be noted that the trace element response to burn differs among studies [42,43,44,45]. For example, studies reported a decrease in serum copper and Cp oxidase activity after major burn injury [18,44] that was little improved by copper supplementation [18], while another observed no change in serum zinc and copper levels [45]. Several post-translational changes in Cp have been shown to affect its activity. Nitration of Cp and oxdative changes have both been shown to decrease the ferroxidase activity [46,47]. Complex formation of Cp with myeloperoxidase and lactoferrin can decrease the oxidase activity and increase the ferroxidase activity of Cp [48,49].

Decreased activity of Cp could potentially have detrimental effects even in the absence of “iron rich” therapeutic interventions because Cp has been identified as a superoxide dismutase mimic which is able to protect against superoxide radicals [50]. Several studies have reported increased oxidative stress and reduced antioxidant defenses in association with trauma and burns [33,34,43,51,52,53,54,55,56]. In our preliminary study with burn patients, although we did not observe variation in serum glutathione peroxidase activity compared to control activity, serum total antioxidant potential was below normal levels for the full 14 day study. In addition, although serum uric acid levels were within the normal range, levels began to fall after burn injury and were lower than admission levels on day 14. Multiple pathways may contribute to the oxidative stress including disrupted iron handling.

This study has some limitations not uncommon in clinical studies. Initial blood samples were not drawn until patients were transferred to the ICU from the emergency department. We had no access to individual patient data to determine who may have received blood transfusions and lastly, patients were enrolled in the study if at least a three day stay in the ICU was anticipated, suggesting a selection bias towards enrolling the sickest patients.

In summary, the data from the present study further support the critical effects of iron metabolism, Cp, oxidative stress and inflammation in traumatic injury states. Further efforts should focus on the role of Cp in regulating iron metabolism after trauma and determine whether Cp oxidase activity influences the effects of exogenous iron that may be administered with transfusions during trauma resuscitation, particularly from older red blood cells. Although hepcidin levels were not determined in the present studies, its interactions with Cp and its role in helping regulate iron metabolism and inflammation associated with trauma [16] should be explored further.

## 5. Conclusions

This study further established the hypoferremia and inflammation associated with burn and non-burn traumatic injury. To our knowledge it is the first study to show an early decrease in Cp oxidase activity in these patient cohorts and support the hypothesis that transient loss of Cp activity contributes to the hypoferremia and inflammation. Considering the role of Cp in iron metabolism regulation, reduced Cp oxidase activity could increase the risk of iron-mediated injuring following therapeutic interventions such as transfusions with aged blood near its 42 day storage limit.
